# Modeling Peak Expiratory Flow in Patients With Asthma and Quantifying Treatment Effects Using a Mixed‐Effects Hidden Markov Model

**DOI:** 10.1002/psp4.70281

**Published:** 2026-06-06

**Authors:** Ludvig Jakobsson, Marcus Baaz, Jacob Leander, Philip Gerlee, Mats Jirstrand

**Affiliations:** ^1^ Department of Mathematical Sciences Chalmers University of Technology and University of Gothenburg Gothenburg Sweden; ^2^ Fraunhofer‐Chalmers Research Centre for Industrial Mathematics Gothenburg Sweden; ^3^ Clinical Pharmacology and Quantitative Pharmacology, Clinical Pharmacology and Safety Sciences, R&D AstraZeneca Gothenburg Sweden; ^4^ Department of Electrical Engineering Chalmers University of Technology Gothenburg Sweden

**Keywords:** dose response, mathematical modeling, mixed effects models, model‐based drug development, respiratory

## Abstract

Clinical trials in asthma and chronic obstructive pulmonary disease often use exacerbation risk as the primary endpoint. However, exacerbations occur with low frequency, leading to long and costly clinical trials. Home‐measured spirometry, which is becoming more common, provides an alternative and has previously been used to shorten the necessary trial duration. In this work, we develop a *mixed‐effects hidden Markov model* (MEHMM) for analyzing home‐measured *peak expiratory flow* (PEF), combining an observation model with a latent two‐state disease process representing sustained periods of high and low PEF, respectively. An inference framework is implemented to estimate fixed and random effects together with measures of uncertainty. Data from a phase 2b dose‐finding study of velsecorat in asthma are used to investigate dose–response relationships, complemented by an extensive simulation study. The results demonstrate reliable estimation of parameters and identify statistically significant treatment effects on multiple model components. These findings support the use of latent disease‐state models for extracting meaningful information from home‐measured spirometry.

## Study Highlights

**What Is the Current Knowledge on the Topic?:**

Lung function variability of patients with asthma fluctuates substantially over time, and increased variability has been linked to an elevated risk of worsening events. Home‐measured spirometry can provide rich temporal data, but its irregular dynamics make it difficult to analyze using conventional modeling approaches.

**What Question Did This Study Address?:**

Can a mixed‐effects hidden Markov model be used to model home‐measured peak expiratory flow (PEF) in asthmatic patients, and can any treatment effects be estimated using a modified stochastic approximation expectation–maximization algorithm (SAEM)?

**What Does This Study Add to Our Knowledge?:**

A mixed‐effects hidden Markov model was developed and used to analyze peak expiratory flow measurements in a clinical trial of patients with asthma. The results showed that parameter estimation and state‐transition estimation were reliable and that several treatment effects were statistically significant. This serves as a proof of concept for modeling PEF using latent state‐dependent variables, which are estimated using a modified SAEM algorithm.

**How Might This Change Clinical Pharmacology or Translational Science?:**

The results add to the broader knowledge on variability in lung function in respiratory diseases, and on the utilization of high‐resolution home‐measured spirometry data. This has the potential to support clinical drug development through the refinement or development of respiratory endpoints.

## 1 Introduction

In respiratory diseases, such as asthma and chronic obstructive pulmonary disease (COPD), lung function variability has been shown to [1, 2] be associated with the risk of acute worsening events, known as exacerbations. These events, during which patients experience increased symptoms and obstructed airflow, account for much of the mortality and treatment costs associated with respiratory diseases [3, 4], and are often chosen as the primary endpoint in late‐stage clinical trials [5]. The incidence of exacerbations is low [6], leading to long and expensive clinical trials. This has prompted efforts to make fuller use of the available lung function data to shorten trial durations while preserving a strong link to exacerbation risk. Clinical advancements have supported these efforts with home‐measured spirometry, providing higher‐resolution data. Detrended fluctuation analysis has previously been used to study the association between variability in home‐measured peak expiratory flow (PEF) measurements and exacerbation risk [1, 7]. More recent work used a stochastic mixed effects model to quantify the effect of various sources of PEF variability on exacerbation risk and found that long‐term variability, as opposed to day‐to‐day variability, was associated with both treatment effect and exacerbation risk [8].

Other recent advances in clinical drug development for respiratory diseases are the event endpoints CompEx Asthma [9] and COPDCompEx [10]. These events are defined by the occurrence of a diary event based on a change from baseline PEF and reliever use, along with other symptom variables. The findings showed that CompEx Asthma resulted in 2.8 times more events than exacerbations and allowed for a 50% reduction in the number of patients needed, while allowing for a 3‐month trial to evaluate the drug effect on exacerbation risk instead of the common study length of 12 months [9]. This has led to CompEx being used as a primary endpoint in several Phase 2 clinical trials in asthma and COPD (NCT06529419, NCT06376045, NCT05492877, NCT05251259) [11, 12, 13, 14].

Our work investigates whether discrete, sustained worsening events can be identified from PEF time series at the individual level, rather than being defined by predetermined thresholds as in the PEF‐component of the CompEx event. This is done by modeling PEF with a discrete‐time mixed‐effects hidden Markov model (MEHMM) [15]. In a paper by Delattre and Lavielle, an inference scheme was proposed for estimating population parameters, individual parameters, and the most likely path of the latent process for each individual in an MEHMM [16]. That approach adapts the standard nonlinear mixed‐effects (NLME) framework by modifying the stochastic approximation expectation–maximization (SAEM) [17] algorithm so that observations from a hidden Markov model (HMM) may be considered. The implementation of the proposed scheme used a combination of the Monolix software and MATLAB to perform the estimations.

We modeled PEF using an MEHMM with inference based on the proposed scheme. Our primary goal was to estimate treatment effects from disease‐state dynamics, rather than modeling the full complexity of PEF trajectories. Accordingly, transitions were assumed to be instantaneous, providing a simplified representation at the observation level. A more extensive discussion on this assumption is given in Section 4. The model's robustness to different data scenarios was tested through a simulation study before data from a phase 2b clinical trial in asthma were analyzed.

The model and the corresponding inference algorithms were implemented in R, and the code is available in a public repository on GitHub (see Supporting Information). The implementation allows for common dose–response relationships to be included directly into the model, making inference about treatment effects practical and straightforward.

The article is organized as follows: First, the modeling framework and the estimation scheme are described in Sections 2.1 and 2.2. This is followed by a presentation of the PEF model and the simulation study framework in Sections 2.3 and 2.4, respectively. In Section 3.1, the results from the simulation study are presented, and in Section 3.2, the model is fitted to clinical trial data from a phase 2b study evaluating an asthma treatment. Finally, in Section 4, the results are discussed and summarized.

## 2 Methods

### 2.1 Mixed‐Effects Hidden Markov Models

Hidden Markov models (HMMs) provide a flexible framework for modeling time series data with underlying discrete latent structures. In this setting, the observed data are assumed to arise from an unobserved, discrete‐time Markov process representing latent states, where each state governs the outcome of observations through a state‐dependent observation distribution. Mixed‐effects hidden Markov models (MEHMM) [15] were defined as an extension of HMMs to include random effects for population approaches, allowing for between‐subject variability in both transition dynamics and observation distributions. This extension is particularly important when individual time series provide limited information for subject‐specific HMM inference, e.g., when no state transitions are observed. Covariates can be incorporated into MEHMMs to alter transition probabilities or observation distributions, enabling the models to account for both individual heterogeneity and treatment effects.

**FIGURE 1:**
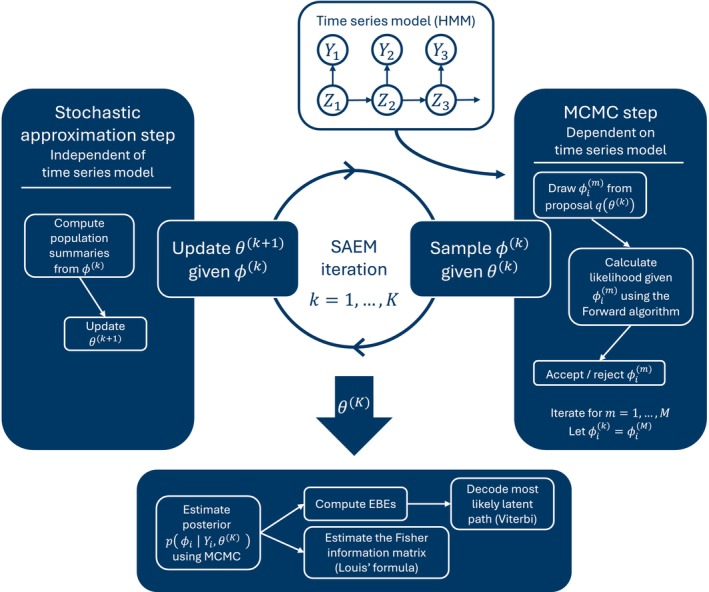
A schematic view of the estimation framework used in this work. The algorithm alternates between a Markov chain Monte Carlo (MCMC) step where patient‐specific parameters $\phi_{i}^{\left(k\right)}$ are sampled conditioned on the current population parameter estimates $\theta^{\left(k\right)}$ and the patient's time series data, and a stochastic approximation step where population summaries are computed from the sampled $\phi_{i}^{\left(k\right)}$ and used to compute $\theta^{\left(k+1\right)}$. After convergence, the final $\hat{\theta }=\theta^{\left(K\right)}$ are used to compute empirical Bayes estimates, estimate the Fisher matrix, and decode the most likely latent path.

For a standard HMM, we let $Y_{i}=\left(Y_{i,1},\ldots ,Y_{i,T}\right)$ be the sequence of observed data for individual i, taking on discrete or continuous values, and let $Z_{i}=\left(Z_{i,1},\ldots ,Z_{i,T}\right)$ be the corresponding latent state sequence with $Z_{i,t}$ evolving with subject‐specific transition probabilities

$$p_{i,j,l}=P\left(Z_{i,t}=l|Z_{i,t-1}=j\right)$$

where j,l=0,…,L represent the possible latent states. The observations ${\left\{Y_{i,t}\right\}}_{t=1}^{T}$ are then assumed conditionally independent given the latent state, with an observation distribution $P\left(Y_{i,t}|Z_{i,t}\right)$ that may depend on other parameters as well. A schematic view of an HMM is shown at the top of Figure 1.

In the extension to MEHMMs, individual heterogeneity is introduced by allowing the parameters of the HMM to vary across individuals via random effects. Let $\psi_{i}$ denote the vector of individual‐specific parameters for individual i, including transition probabilities and any parameters of the observation distribution. Assume that we have a transformation g so that $\phi_{i}=g\left(\psi_{i}\right)$ is Gaussian in each component. The transformed parameters $\phi_{i}$ are then defined as

$$\phi_{i}=\mu +\beta \left(x_{i}\right)+\eta_{i}$$

where $\eta_{i}∼N\left(0,\Omega \right)$ is the vector of random effects with covariate matrix Ω, μ is the vector of population reference values, and $x_{i}$ is a vector of covariates giving effects $\beta \left(x_{i}\right)$. The population parameters of the model are denoted $\theta =\left(\mu ,\beta ,\Omega \right)$, with β containing all covariate parameters.

### 2.2 Estimation Scheme

When estimating the parameters of a standard HMM, a version of the expectation–maximization (EM) algorithm is most used. This version is often referred to as the Baum‐Welch algorithm [18] and utilizes the forward‐backward algorithm [18] in the expectation step to solve the smoothing problem, which is to compute $P\left(Z_{t}|Y_{1:T}\right)$, for all t=1,…,T.

Building on the MEHMM framework and the Baum–Welch algorithm, an estimation scheme [16] was proposed using the stochastic approximation expectation–maximization algorithm (SAEM) [17]. The method enables the estimation of both fixed and random effects, including covariate effects, as well as individual‐specific empirical Bayes estimates (EBE) along with latent process decoding via the Viterbi [18] algorithm and estimation of standard errors. The following notes on the method are included for the convenience of the reader.

The standard SAEM algorithm for NLME models requires a slight modification to work with MEHMMs. In the simulation step, the goal is to sample from the posterior distribution $P\left(\phi_{i}|Y_{i},\theta^{\left(k\right)}\right)$, where k=1,…,K is the current iteration of the algorithm. When sampling from the posterior distribution is intractable, samples are often drawn via a Markov chain Monte Carlo (MCMC) method, which requires evaluation of the full likelihood

$$P\left(Y_{i},\phi_{i}^{\left(m\right)}|\theta^{\left(k\right)}\right)=P\left(Y_{i}|\phi_{i}^{\left(m\right)},\theta^{\left(k\right)}\right)\times P\left(\phi_{i}^{\left(m\right)}|\theta^{\left(k\right)}\right)$$

for each set of proposed parameters $\phi_{i}^{\left(m\right)}∼q\left(\theta^{\left(k\right)}\right)$, m=1,…,M, given a proposal density q. In classical NLME models, this can be evaluated analytically. However, for MEHMMs, evaluation of the first factor above requires the use of the forward recursions of the forward‐backward algorithm since the latent process is unknown. This modification is straightforward when implementing the SAEM algorithm manually, but requires workarounds when using existing software.

The likelihood calculation is simplified by noting that $Y_{i}$ is conditionally independent of $\theta^{\left(k\right)}$ given $\phi_{i}^{\left(m\right)}$, that is

$$P\left(Y_{i}|\phi_{i}^{\left(m\right)},\theta^{\left(k\right)}\right)=P\left(Y_{i}|\phi_{i}^{\left(m\right)}\right)$$

This further simplifies the maximization step of the SAEM algorithm and the approximation of the standard errors since only the second factor of the likelihood now depends on the population parameters θ, and $P\left(\phi_{i}^{\left(m\right)}|\theta^{\left(k\right)}\right)$ is a known distribution.

Standard errors can be calculated from the obtained population parameter estimates $\hat{\theta }$ using Louis' formula to estimate the Fisher information matrix [19, 20]. The expected values present in Louis' formula are estimated by running an MCMC chain to approximate the posterior distribution of each parameter given the final population parameter estimates $\hat{\theta }$. The entire algorithm is described schematically in Figure 1.

### 2.3 Modeling Peak Expiratory Flow

Here, we will present an MEHMM for modeling PEF measurements in respiratory diseases. Building on the framework presented above, we assume that each patient's observed PEF depends on a latent process with two states, *high PEF* and *low PEF*, representing the patient being in a normal health condition and in a worsened health condition, respectively, as well as several individual‐specific model parameters following some population distribution. The patients are assumed to be given either a placebo drug or an active drug at some dose $x_{i}$. The treatment effect is included in the model as a dose‐dependent additive effect on each transformed parameter, as in Section 2.1. Dose was selected as the exposure variable since, for inhaled therapies, the pharmacologically relevant exposure is commonly the local drug concentration in the lung and not plasma concentration [21]. However, the model could be modified to consider fuller pharmacokinetic‐pharmacodynamic relationships, including, e.g., a delayed response and other routes of administration.

The PEF measurement $Y_{i,t}$ and the state transition probabilities for subject i=1,…,N at time t=1,…,T are modeled as

$$Y_{i,t}=m_{i,Z_{i,t}}+\varepsilon_{i,t},$$

$$P\left(Z_{i,t}=l|Z_{i,t-1}=j\right)=p_{i,j,l},$$

where $\varepsilon_{i,t}∼N\left(0,\sigma_{i}^{2}\right)$ and $j,l\in \left\{0,1\right\}$, with individual‐specific model parameters

$$\psi_{i}=\left(m_{i,0},d_{i},\sigma_{i}^{2},p_{i,0,1},p_{i,1,0}\right).$$

The parameter $d_{i}\in \left[0,1\right]$ defines the relative drop from high PEF $m_{i,0}$ to low PEF $m_{i,1}=d_{i}\times m_{i,0}$. Note that the pair $\left(p_{i,0,1},p_{i,1,0}\right)$ fully defines the transition matrix $P_{i}$ for individual i since each row sums to one. The individual parameters are in turn transformed using a transformation $\phi_{i}=g\left(\psi_{i}\right)$ that applies logarithmic or logit functions depending on the parameter and are then modeled as:

$$\log\left(m_{i,0}\right)=\mu_{1}+\beta_{1}\left(x_{i}\right)+\eta_{1,i}$$

$$\text{logit}\left(d_{i}\right)=\mu_{2}+\beta_{2}\left(x_{i}\right)+\eta_{2,i}$$

$$\log\left(\sigma_{i}^{2}\right)=\mu_{3}+\beta_{3}\left(x_{i}\right)+\eta_{3,i}$$

$$\text{logit}\left(2p_{i,0,1}\right)=\mu_{4}+\beta_{4}\left(x_{i}\right)+\eta_{4,i}$$

$$\text{logit}\left(2p_{i,1,0}\right)=\mu_{5}+\beta_{5}\left(x_{i}\right)+\eta_{5,i}$$

where $\eta_{c,i}∼N\left(0,\omega_{c}^{2}\right)$ for each i=1,…,N and c=1,…,5. The factor 2 in the definitions of $p_{i,0,1}$ and $p_{i,1,0}$ restricts these parameters to the interval $\left[0,0.5\right]$, reflecting the assumption of sustained rather than rapid transitions, which is consistent with the aim of this work. The random effects are assumed to be independent across the components of $\phi_{i}$, meaning that the covariate matrix Ω is diagonal. This simplifies the MCMC algorithm since the samples can be drawn independently across parameters, but a full covariance matrix could also be considered.

Four types of dose–response relationships were implemented in the code:

$$\text{Categorical}\;\beta \left(x_{i}\right)=\sum \beta_{p}\left(x_{i,p}\right)$$

$$\text{Constant}\;\beta \left(x_{i}\right)=\beta 1_{\left\{x_{i}>0\right\}}$$

$$\text{Linear}\;\beta \left(x_{i}\right)=\beta x_{i}$$

$$\text{Emax}\;\beta \left(x_{i}\right)=\frac{E_{\max}x_{i}}{ED_{50}+x_{i}}$$

where $x_{i}$ is a continuous dose in the constant, linear, and Emax models, while $x_{i}=\left(x_{i,1},\ldots ,x_{i,P}\right)\in {\left\{0,1\right\}}^{P}$ with $\sum_{p}x_{i,p}=1$ is a categorical dose in the categorical model. To explore treatment effects on each parameter, we first fit a flexible model using categorical treatment indicators, henceforth denoted the exploratory model. This is analogous to an ANOVA‐type analysis. After running the estimation scheme, non‐significant treatment effects were removed to obtain a more parsimonious model. Finally, based on the pattern observed in the exploratory step, we evaluated parametric dose–response models.

### 2.4 Simulation Study

Datasets were simulated in R using the PEF model above with population parameter values chosen to reproduce the mean and variance of twice‐daily home‐measured PEF observed in clinical trials. A similar number of patients per treatment group and similar time series lengths were also used. One active treatment arm was simulated alongside a placebo, and a categorical treatment effect was used for all parameters. The algorithms described in Section 2.2 were then used for the estimation of parameters from the simulated datasets.

Given the point estimates $\hat{\theta }$ of fixed and random effects in each run, 200 samples from the posterior distribution $P\left(\phi_{i}|y_{i},\hat{\theta }\right)$ were drawn for each subject i using the MCMC algorithm. These samples were then used to calculate EBEs as the means of the posterior samples and to estimate the Fisher information matrix. The EBEs were, in turn, used to calculate the most likely latent path via the Viterbi algorithm.

The point estimates $\hat{\theta }$ were assessed by bias, calculated as the average difference between the estimates and the true parameters used to simulate the data. The EBEs were evaluated by their predictive accuracy with respect to the true simulated individual parameters and by the η‐shrinkage [22]. The accuracy of the paths generated by the Viterbi algorithm was measured by confusion matrices for each simulated dataset. Finally, the standard error estimates were assessed per‐parameter by their average difference from the standard deviation of the parameter estimates $\hat{\theta }$ and by the coverage of 95%‐confidence intervals for $\hat{\theta }$.

### 2.5 Clinical Trial Data

Patient‐level data from a phase 2b dose‐finding study of velsecorat in asthmatic patients (NCT03622112) [23] was used in model development and in the analysis. The clinical trial featured 5 active treatment arms and a placebo group with twice‐daily home‐measured PEF collected during a 12‐week treatment period. Before the analysis, all informed consent forms were reviewed for data reuse in accordance with AstraZeneca data‐sharing rules.

## 3 Results

### 3.1 Simulation Study

The results from 200 simulated datasets are summarized in Table 1 which shows the true parameter values, the mean and standard deviation of the population parameter estimates, the mean standard error estimates, and the coverage of the 95% confidence intervals calculated from the per‐dataset estimates. The results are shown for models with a categorical treatment effect on all parameters and datasets with N=100 subjects per treatment group and timeseries lengths T=200. The analysis was also carried out for datasets with timeseries lengths T=50 and T=1000, respectively, as reported in Tables S1 and S2 in the Supporting Information. For the time series of length T=200, all estimates converged, and the coverage ranged between 91%–97%, with a mean of 94%.

**TABLE 1:** Summarized results from estimating parameters using simulated data sets of length T=200 with one active treatment group and a placebo group. The treatment effects β are categorical and additive on the transformed individual‐specific parameters for the active group.

Parameter	True value	Mean estimate	Standard deviation of estimate	Mean standard error	Coverage (%)
High PEF $m_{0}$					
$\mu_{1}$	5.521	5.526	0.030	0.032	97
$\beta_{1}$	0.250	0.246	0.043	0.045	96
$\omega_{1}^{2}$	0.100	0.101	0.010	0.010	95
Drop d					
$\mu_{2}$	1.000	0.994	0.054	0.050	91
$\beta_{2}$	0.000	0.001	0.076	0.072	93
$\omega_{2}^{2}$	0.250	0.250	0.026	0.026	96
Inter‐state variability $\sigma^{2}$					
$\mu_{3}$	5.704	5.702	0.024	0.025	96
$\beta_{3}$	0.000	0.004	0.034	0.035	96
$\omega_{3}^{2}$	0.050	0.051	0.007	0.006	94
Transition probability $p_{0,1}$					
$\mu_{4}$	−2.197	−2.181	0.086	0.080	93
$\beta_{4}$	−0.500	−0.527	0.122	0.115	94
$\omega_{4}^{2}$	0.400	0.388	0.071	0.068	92
Transition probability $p_{1,0}$					
$\mu_{5}$	−2.197	−2.203	0.081	0.077	94
$\beta_{5}$	0.000	−0.003	0.124	0.111	92
$\omega_{5}^{2}$	0.300	0.312	0.072	0.062	92

The average per‐parameter relative difference between the true individual parameters and the EBEs across all simulated datasets were small for the parameters $m_{0}$, d, and $\sigma^{2}$, and slightly larger for the transition probabilities (−10% and −12%, respectively). The EBEs are plotted against the true individual parameters, together with a coefficient of determination ($R^{2}$) corresponding to a linear regression, in Figure S1 in the Supporting Information. Figure S2 shows the shrinkage in each model parameter by comparing the estimated population variance to the sample variance of the EBEs.

**FIGURE 2:**
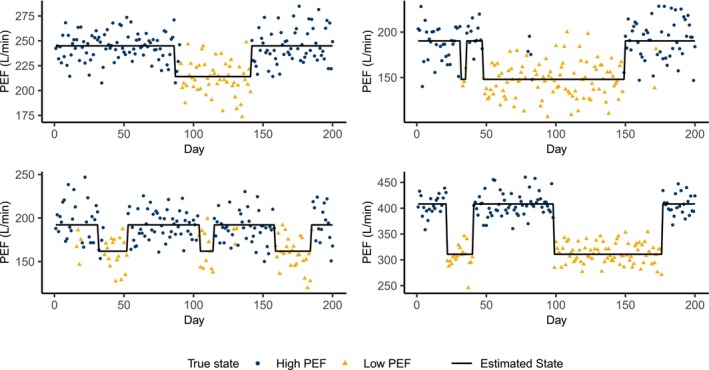
Four simulated individual time series from a mixed‐effects hidden Markov model with the population parameters from Table 1. The true latent state for each observation is indicated by the point color, and the estimated most likely latent path from the fitted model is shown as an overlaid line. The y‐position of the line corresponds to the estimated mean of each state.

Figure 2 shows four typical simulated time series colored by true latent states and estimated latent‐state paths. Finally, given each subject's time series and latent‐state path, the average accuracy over all data sets, calculated as the sum of the diagonal of the average confusion matrix, was 98.9%.

### 3.2 Application to Clinical Trial Data

The parameter estimates from the exploratory model with categorical treatment effects are shown in Table S3 in the Supporting Information along with the estimated standard errors. This model allowed each treatment group to have its own effects and made no assumptions about dose–response relationships. Several treatment groups showed statistically significant effects vs. placebo for the drop parameter d, the inter‐state variability $\sigma^{2}$, and the transition probability $p_{1,0}$. The treatment effects for the remaining parameters $m_{0}$ and $p_{0,1}$ were subsequently removed before dose–response models were considered.

**FIGURE 3:**
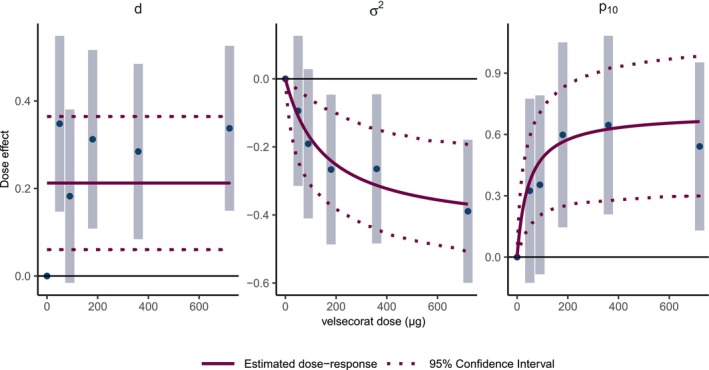
Estimated dose effect for the drop parameter d, the inter‐state variability $\sigma^{2}$, and the transition probability $p_{1,0}$. The fitted dose–response curve (constant effect for d, and an Emax model for $\sigma^{2},\text{and}\;p_{1,0}$) is shown as a solid line with 95%‐confidence interval as dotted lines. The points and corresponding bars indicate the estimated dose effect in each treatment group from the exploratory model.

Based on visual inspection, an Emax model was selected and fitted for the parameters $\sigma^{2}$ and $p_{1,0}$, whereas a constant effect was assumed for the parameter d, giving equal effect for all active treatment groups and no effect for the placebo group. The resulting fitted dose–response curves are overlaid with the categorical treatment effects obtained in the exploratory model and are shown in Figure 3. For the Emax dose–response curves, confidence intervals were obtained by sampling parameters using the estimated covariance matrix and computing the 2.5% and 97.5% quantiles for the response. For the constant dose–response curve and the categorical treatment effects, confidence intervals were calculated from the corresponding estimated standard errors. All population parameter estimates and standard errors for this model are given in Table S4 in the Supporting Information. Given these estimates, the final mean parameter values, which have been transformed using $g^{-1}\left(\phi \right)$, for the placebo group and the highest dose group are given in Table 2, along with 95% confidence intervals.

**TABLE 2:** Mean model parameters in the placebo group and the highest‐dose treatment group, with 95%‐confidence intervals.

Parameter	Placebo	720 μg
$m_{0}$	317 (309, 326)	317 (309, 326)
d	0.879 (0.863, 0.893)	0.900 (0.895, 0.905)
σ	30.5 (28.4, 32.8)	25.4 (24.5, 27.4)
$p_{0,1}$	0.031 (0.028, 0.034)	0.031 (0.028, 0.034)
$p_{1,0}$	0.023 (0.017, 0.031)	0.043 (0.037, 0.050)

Akaike's information criteria (AIC) for this model (denoted as ‘mixed’) was compared to the AIC of the exploratory model (‘categorical’) and a model without any dose effects (‘none’). This is shown in Table S5 in the Supporting Information along with the $R^{2}$ of a linear regression between the PEF measurements and the predictions obtained for each model. Furthermore, Figure S3 shows the distribution of the estimated random effects obtained from the mixed dose–response model.

**FIGURE 4:**
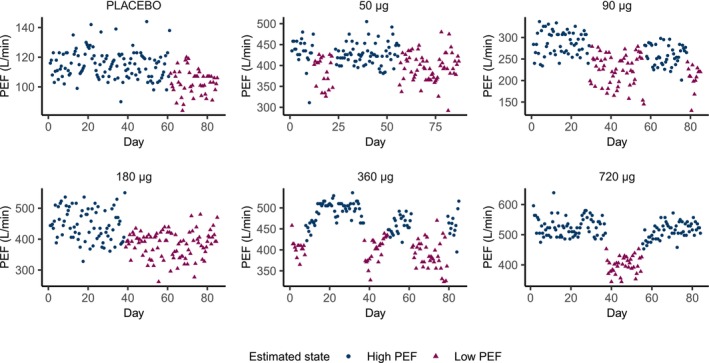
Six individual PEF time series from the clinical trial, one from each treatment group. The most likely latent path, estimated using the Viterbi algorithm and the individual empirical Bayes estimates, is indicated by the point color and shape.

**FIGURE 5:**
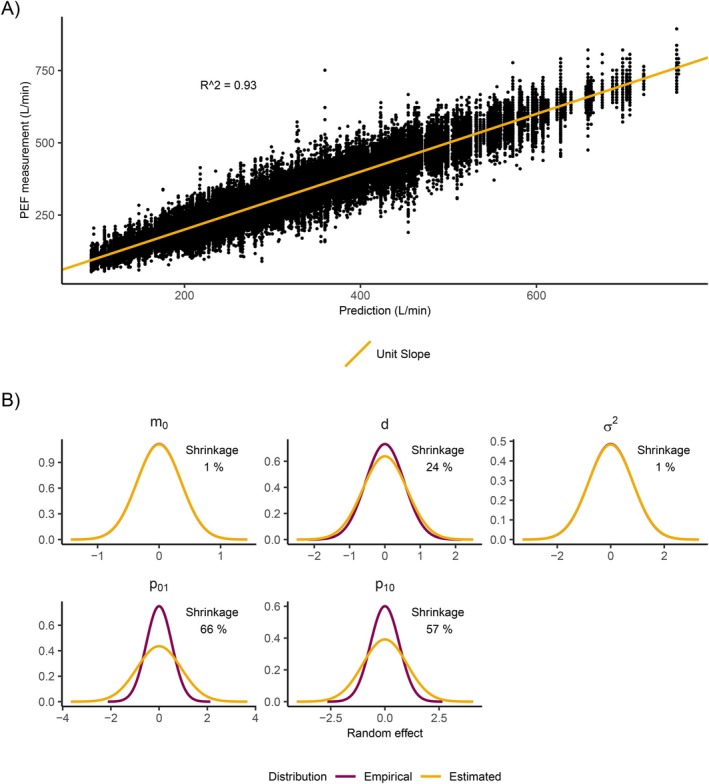
(A) Individual PEF predictions plotted against PEF measurements with a unit slope line and the coefficient of determination ($R^{2}$) of a linear regression. (B) Distribution plots showing η‐shrinkage for each model parameter in the clinical trial. The estimated distributions have variance equal to the estimated population variance $\omega_{c}^{2}$ for c=1,…,5 and the empirical distributions have variance equal to the sample variance of the individual empirical Bayes estimates.

Given the mixed dose–response model, PEF time series with their estimated most likely latent paths, calculated using the EBEs, are shown in Figure 4 for selected individuals. Some shrinkage was observed in the EBEs, mainly for the transition probabilities (η‐shrinkage 66% and 57%, respectively), which is illustrated in Figure 5 by the estimated population distributions along with the empirical distributions obtained from the EBEs. Figure 5 also shows predicted PEF plotted against the PEF measurements together with the $R^{2}$ of a linear regression between the two.

## 4 Discussion

This work considered an application of the SAEM algorithm adapted to handle observations described by an MEHMM, as proposed by Delattre and Lavielle [16]. A two‐state Gaussian MEHMM was developed to model home‐measured PEF with potential treatment effects on all model parameters. The implementation of the estimation scheme was verified in a simulation study and subsequently applied to home‐measured spirometry data from a clinical trial of patients with asthma to evaluate its ability to distinguish treatment effects. The results of the simulation study showed that the estimation scheme produces estimates of fixed and random effects with low bias and standard errors that reflect the variability in parameter estimates obtained from repeated simulations of datasets. Estimation of individual‐specific EBEs was found to reflect the true simulated parameters to some degree, but shrinkage was observed. This indicates limited patient‐level identifiability for time series of length T=200 and motivates the use of random effects to get reliable population parameter estimates. The most likely latent path, estimated using the Viterbi algorithm, was compared to the true latent path by a mean confusion matrix over all simulated datasets and by visual inspection of latent paths plotted together with simulated data. Figure 2 shows how some short state transitions are difficult to estimate, but overall, the results show a reliable estimation of state transitions given EBEs, despite the observed shrinkage.

The application of the model and estimation scheme to home‐measured PEF time series data shows promising results. Overall, the obtained parameter estimates were in a reasonable range, and the relative standard errors were in most cases small. Figure 4 shows that the inferred latent paths broadly follow the expected transition dynamics with sustained periods in both states. Furthermore, statistically significant treatment effects were found for several treatment groups for the drop parameter d, the inter‐state variability $\sigma^{2}$, and the transition probability $p_{1,0}$ without any assumptions on the dose–response relationship. These treatment effects were consistent with the expected direction of effect since a higher value of the parameter d indicates less severe worsened states, a lower value of the parameter $\sigma^{2}$ indicates more stable measurements within each state, and a higher value of the parameter $p_{1,0}$ indicates faster recovery from the low PEF state. No discernible effect was observed on the parameter $m_{0}$, despite prior expectation that the treatment would increase average PEF. A possible explanation for this is the substantial dropout observed in the placebo group of the clinical trial, which may have inflated the estimated mean value of $m_{0}$, thus underestimating the dose effects. These cases of informative censoring may be addressed in future work by including a risk model of early discontinuation in the PEF model presented here and estimating all parameters jointly, similar to the approach of Król et al. [24]. Another explanation for the lack of treatment effect is the assumption of an instantaneous effect following randomization. In reality, the effect is likely to increase gradually over the course of the study until it reaches its maximum. An extension of our work could consider a turnover model for one or several treatment effects to account for this delay, see, e.g., Leander et al. [8].

Two constraints were imposed on the individual parameters: An upper limit of 0.5 for the transition probabilities and a diagonal covariance matrix Ω. The upper limit was imposed to avoid unrealistically rapid transitions driven by outlier measurements. Without this constraint, transition probabilities were at times estimated close to one, corresponding to near‐instant switches between the states. The independence between parameters was chosen for ease of implementation. However, moderate correlations were observed between the EBEs of the parameters $m_{0}$, d, and $\sigma^{2}$, suggesting that the covariance structure may be somewhat restrictive and that allowing for correlations in selected parameters could provide a more realistic representation of the inter‐individual variability.

The parameters $\sigma^{2}$ and $p_{1,0}$ showed clear signs of saturation in the exploratory model, motivating the use of an Emax dose–response model. The resulting fitted curves reproduced the categorical estimates closely and with reduced uncertainty. However, while the Emax parameter estimates were precise, the standard errors of the ED50 parameter estimates were substantial. This is expected when the available dose levels do not adequately characterize the steep part of the dose–response curve, but it nonetheless reduces confidence that Emax is the most appropriate representation of the underlying dose effect. Despite this, the model selection criteria AIC favored this model over the model without treatment effects and the model with categorical treatment effects. Furthermore, the estimated random effects from this model, stratified on treatment group, were centered at zero for all model parameters.

Future work could extend the MEHMM model to include a temporal dependence in the observation model, e.g., by modeling the observations as an autoregressive or stochastic process [8]. This would allow for smoother state transitions on the observation level, which are present in the data in Figure 4, instead of the assumed instant transitions considered in this work, and may offer more insight into the role of discrete states in lung function. Such an extension would require another method for computing the likelihood on the patient level, but would otherwise be straightforward to include within the provided implementation of the SAEM algorithm. A final natural extension is to broaden the model beyond only PEF time series. Other variables, such as daily patient‐reported symptom scores and rescue medication use, are known to be affected by treatment and are informative of patient health [25, 26]. Future work could therefore extend the model to include these variables alongside PEF.

Overall, the results show potential for modeling home‐measured PEF using a latent time‐dependent discrete disease‐state variable with the possibility to include treatment effects in the model parameters. If the proposed model is to support clinical decision making, additional work needs to determine whether the estimated treatment effects in the parameters correlate with other clinical outcomes in asthma, for instance, the risk of experiencing an exacerbation or a CompEx event.

## Author Contributions

L.J., M.B., J.L., P.G., and M.J. wrote the manuscript; L.J., M.B., J.L., P.G., and M.J. designed the research; L.J. and J.L. performed the research; L.J. analyzed the data.

## Funding

The work was funded by AstraZeneca.

## Conflicts of Interest

J. Leander is an employee of AstraZeneca and may own stock/stock options. All other authors declared no competing conflicts of interest for this work.

## Data Availability Statement

R code sufficient for performing the simulation and estimation in this work is publicly available at https://github.com/fcc‐ludvig‐jakobsson/pef‐mehmm.git.

## Supporting information


**Figure S1:** Individual empirical Bayes estimates (EBE) plotted against true simulated individual parameters with unit slope lines and per‐parameter coefficients of determination (𝑅 2). The points show all pairs of individual parameter values and EBEs from the first 25 simulated data sets.
**Figure S2:** Distribution plots showing 𝜂‐shrinkage for each model parameter across all 200 simulated datasets. The estimated distributions have variance equal to the estimated population variance ωc2𝜔𝑐 2 for 𝑐 = 1,…,5, and the empirical distributions have variance equal to the sample variance of the individual empirical Bayes estimates.
**Table S1:** Summarized results from estimating parameters using simulated data sets of length T = 50. Runs that resulted in failed standard error estimations were excluded from the coverage calculation.
**Table S2:** Summarized results from estimating parameters using simulated data sets of length T = 1000.
**Table S3:** Population parameter estimates from the exploratory model with categorical treatment groups fitted to the clinical trial data.
**Table S4:** Population parameter estimates from the mixed dose–response model fitted to the clinical trial data.
**Table S5:** Comparison of three models incorporating different dose–response relationships using Akaike's information criteria (AIC) and the coefficient of determination (𝑅2) of a linear regression between observed and predicted PEF values. The model denoted none includes no treatment effects; the categorical model includes a categorical effect for every parameter and treatment group; and the mixed model includes a constant treatment effect for the parameter d and Emax dose–response relationships for 𝜎2 and 𝑝1,0. The AIC is computed as 2𝑝 − ln(𝐿 ^), where 𝑝 is the number of parameters in the model, and ln(𝐿 ^) is the estimated log‐likelihood computed from 25,000 posterior samples per individual. Thus, a lower AIC is considered better.
**Figure S3:** Boxplots showing the distribution of estimated random effects from the model denoted “mixed”, per model parameter, and stratified on treatment group. The horizontal line is drawn at 𝑦 = 0.
