# Supplementary material for: Modeling Peak Expiratory Flow in Patients With Asthma and Quantifying Treatment Effects Using a Mixed‐Effects Hidden Markov Model
Source: CPT Pharmacometrics Syst Pharmacol. 2026 Jun 6;15(6):e70281. doi: 10.1002/psp4.70281 (PMC13245144; doi:10.1002/psp4.70281)
Supplement: Supplementary file 1 — Figure S1: Individual empirical Bayes estimates (EBE) plotted against true simulated individual parameters with unit slope lines and per‐parameter coefficients of determination (𝑅 2). The points show all pairs of individual parameter values and EBEs from the first 25 simulated data sets. Figure S2: Distribution plots showing 𝜂‐shrinkage for each model parameter across all 200 simulated datasets. The estimated distributions have variance equal to the estimated population variance ωc2𝜔𝑐 2 for 𝑐 = 1,…,5, and the empirical distributions have variance equal to the sample variance of the individual empirical Bayes estimates. Table S1: Summarized results from estimating parameters using simulated data sets of length T = 50. Runs that resulted in failed standard error estimations were excluded from the coverage calculation. Table S2: Summarized results from estimating parameters using simulated data sets of length T = 1000. Table S3: Population parameter estimates from the exploratory model with categorical treatment groups fitted to the clinical trial data. Table S4: Population parameter estimates from the mixed dose–response model fitted to the clinical trial data. Table S5: Comparison of three models incorporating different dose–response relationships using Akaike's information criteria (AIC) and the coefficient of determination (𝑅2) of a linear regression between observed and predicted PEF values. The model denoted none includes no treatment effects; the categorical model includes a categorical effect for every parameter and treatment group; and the mixed model includes a constant treatment effect for the parameter d and Emax dose–response relationships for 𝜎2 and 𝑝1,0. The AIC is computed as 2𝑝 − ln(𝐿 ^), where 𝑝 is the number of parameters in the model, and ln(𝐿 ^) is the estimated log‐likelihood computed from 25,000 posterior samples per individual. Thus, a lower AIC is considered better. Figure S3: Boxplots showing the distribution of estimated random effects from t [file PSP4-15-e70281-s001.pdf]

Supplementary material

“Modeling peak expiratory flow in patients with asthma and quantifying treatment effects using a mixed-effects hidden Markov model”

Ludvig Jakobsson; Marcus Baaz; Jacob Leander; Philip Gerlee; Mats Jirstrand

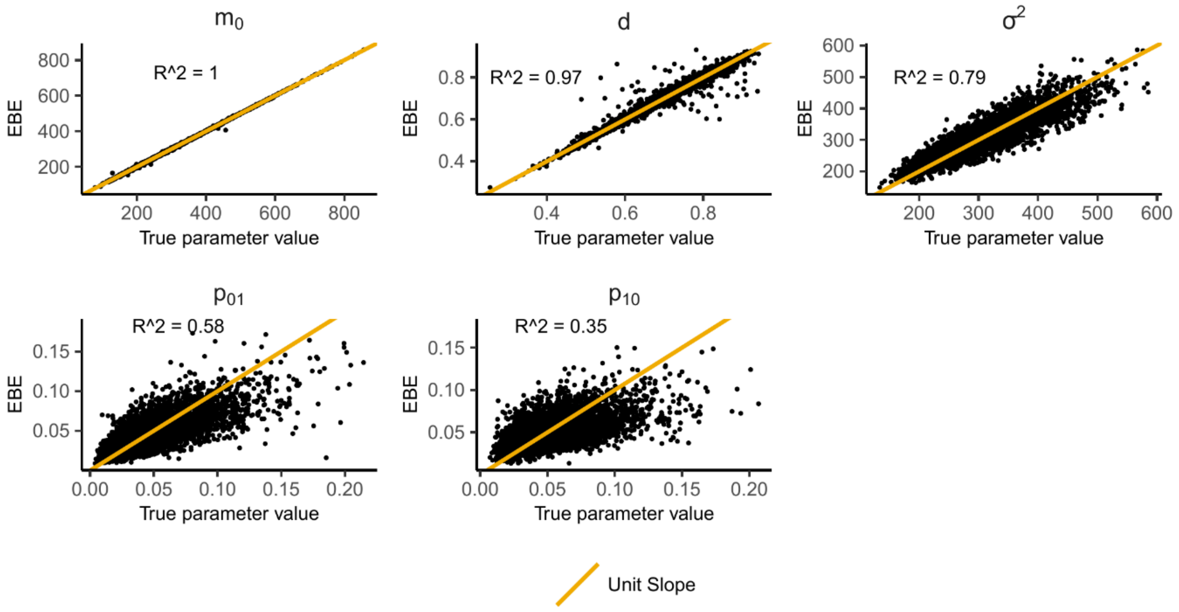

Figure S1: *Individual empirical Bayes estimates (EBE) plotted against true simulated individual parameters with unit slope lines and per-parameter coefficients of determination (R^2). The points show all pairs of individual parameter values and EBEs from the first 25 simulated data sets.*

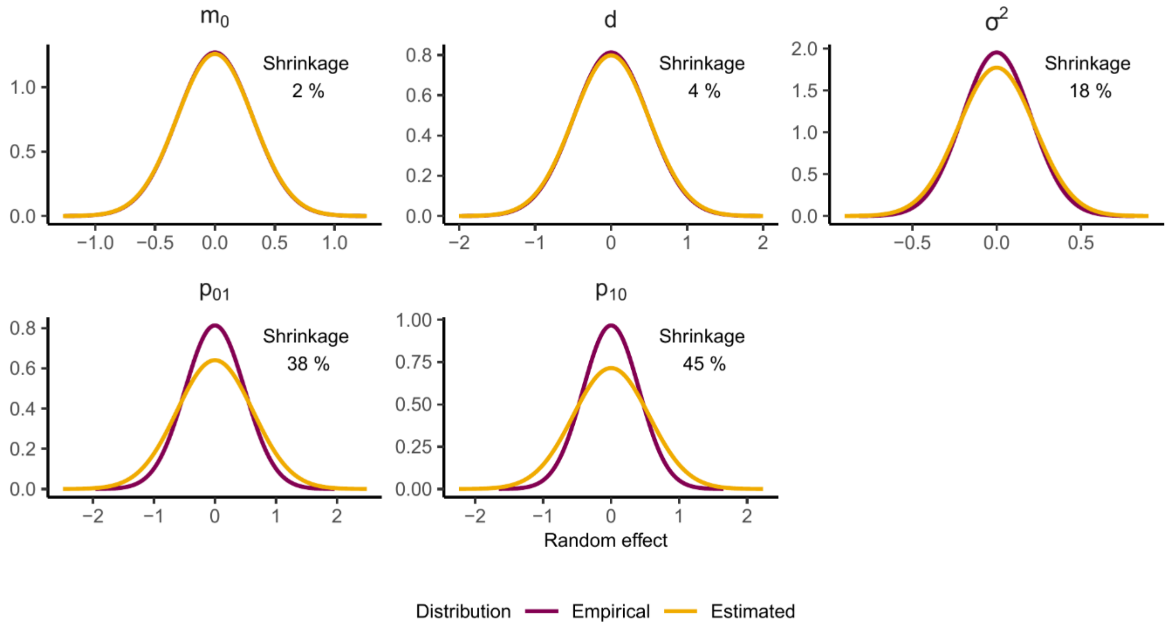

Figure S2: *Distribution plots showing η-shrinkage for each model parameter across all 200 simulated datasets. The estimated distributions have variance equal to the estimated population variance* ω_c^2 *for* $c = 1, \ldots, 5$ *and the empirical distributions have variance equal to the sample variance of the individual empirical Bayes estimates.*

Table S1: *Summarized results from estimating parameters using simulated data sets of length* $T = 50$. *Runs which resulted in failed standard error estimations were excluded in the coverage calculation.*

**Parameter**	**True value**	**Mean estimate**	**Standard deviation of estimate**	**Mean standard error***	**Coverage* (%)**	**Failed SE estimations (%)**
High PEF m_0						
μ_1	5.521	5.526	0.034	0.031	93	0
β_1	0.250	0.247	0.049	0.044	92	0
ω_1^2	0.100	0.096	0.010	0.010	88	0
Drop d						
μ_2	1.000	0.994	0.059	0.055	97	0
β_2	0.000	0.008	0.086	0.082	93	0
ω_2^2	0.250	0.237	0.032	0.030	86	0
Inter-state variability σ^2						
μ_3	5.704	5.706	0.028	0.030	96	0
β_3	0.000	-0.004	0.044	0.042	93	0
ω_3^2	0.050	0.047	0.010	0.009	88	1
Transition probability $p_{0,1}$						
μ_4	-2.197	-2.134	0.139	0.071	54	4
β_4	-0.500	-0.515	0.199	0.107	55	4
ω_4^2	0.400	0.231	0.267	0.091	47	14
Transition probability $p_{1,0}$						
μ_5	-2.197	-2.254	0.154	0.046	37	6
β_5	0.000	0.021	0.246	0.071	35	8
ω_5^2	0.300	0.085	0.172	0.047	21	23

Table S2: *Summarized results from estimating parameters using simulated data sets of length* $T = 1000$.

Parameter	True value	Mean estimate	Standard deviation of estimate	Mean standard error*	Coverage* (%)	Failed SE estimations (%)
High PEF m_0						
μ_1	5.521	5.523	0.032	0.032	96	0
β_1	0.250	0.252	0.046	0.045	95	0
ω_1^2	0.100	0.099	0.010	0.010	93	0
Drop d						
μ_2	1.000	0.998	0.053	0.050	95	0
β_2	0.000	0.002	0.077	0.071	95	0
ω_2^2	0.250	0.250	0.025	0.025	94	0
Inter-state variability σ^2						
μ_3	5.704	5.704	0.023	0.023	94	0
β_3	0.000	0.000	0.032	0.032	96	0
ω_3^2	0.050	0.050	0.005	0.005	97	0
Transition probability $p_{0,1}$						
μ_4	-2.197	-2.197	0.069	0.068	96	0
β_4	-0.500	-0.503	0.093	0.096	96	0
ω_4^2	0.400	0.403	0.057	0.048	92	0
Transition probability $p_{1,0}$						
μ_5	-2.197	-2.199	0.058	0.060	97	0
β_5	0.000	-0.001	0.082	0.086	96	0
ω_5^2	0.300	0.306	0.044	0.038	93	0

Table S3: *Population parameter estimates from the exploratory model with categorical treatment groups fitted to the clinical trial data.*

Parameter	Estimate	Standard error
High PEF m_0		
μ_1	5.745	0.034
$\beta_{1,50}$	0.030	0.048
$\beta_{1,90}$	0.013	0.048
$\beta_{1,180}$	-0.063	0.048
$\beta_{1,360}$	0.040	0.048
$\beta_{1,720}$	0.045	0.046
ω_1^2	0.130	0.007
Drop d		
μ_2	1.992	0.072
$\beta_{2,50}$	0.267	0.100
$\beta_{2,90}$	0.136	0.099
$\beta_{2,180}$	0.283	0.103
$\beta_{2,360}$	0.227	0.100
$\beta_{2,720}$	0.288	0.095
ω_2^2	0.409	0.030
Inter-state variability σ^2		
μ_3	6.837	0.080
$\beta_{3,50}$	-0.084	0.113
$\beta_{3,90}$	-0.179	0.112
$\beta_{3,180}$	-0.250	0.113
$\beta_{3,360}$	-0.256	0.112
$\beta_{3,720}$	-0.370	0.108
ω_3^2	0.683	0.038
Transition probability $p_{0,1}$		
μ_4	-2.806	0.135
$\beta_{4,50}$	-0.119	0.179
$\beta_{4,90}$	0.013	0.185
$\beta_{4,180}$	-0.114	0.195
$\beta_{4,360}$	0.108	0.190
$\beta_{4,720}$	0.117	0.175
ω_4^2	0.563	0.087
Transition probability $p_{1,0}$		
μ_5	-3.026	0.167
$\beta_{5,50}$	0.203	0.226
$\beta_{5,90}$	0.295	0.226

$\beta_{5,180}$	0.633	0.235
$\beta_{5,360}$	0.610	0.221
$\beta_{5,720}$	0.465	0.214
ω_5^2	0.899	0.138

Table S4: *Population parameter estimates from the mixed dose-response model fitted to the clinical trial data.*

Parameter	Estimate	Standard error
High PEF m_0		
μ_1	5.755	0.014
ω_1^2	0.129	0.007
Drop d		
μ_2	1.984	0.072
β_2	0.212	0.078
ω_2^2	0.390	0.027
Inter-state variability σ^2		
μ_3	6.837	0.073
$E_{max,3}$	-0.447	0.133
$\log(ED_{50,3})$	5.039	0.956
ω_3^2	0.681	0.038
Transition probability $p_{0,1}$		
μ_4	-2.716	0.057
ω_4^2	0.839	0.127
Transition probability $p_{1,0}$		
μ_5	-3.008	0.162
$E_{max,5}$	0.705	0.235
$\log(ED_{50,5})$	3.811	0.700
ω_5^2	1.038	0.133

Table S5: *Comparison of three models incorporating different dose-response relationships using Akaike's information criteria (AIC) and the coefficient of determination (R^2) of a linear regression between observed and predicted PEF values. The model denoted* ***none*** *includes no treatment effects; the* ***categorical*** *model includes a categorical effect for every parameter and treatment group; and the* ***mixed*** *model includes a constant treatment effect for the parameter d and Emax dose-response relationships for σ^2 and $p_{1,0}$. The AIC is computed as $2p - \ln(\hat{L})$, where p is the number of parameters in the model and $\ln(\hat{L})$ is the estimated log-likelihood computed from 25000 posterior samples per individual. Thus, a lower AIC is considered better.*

**Dose-response model (# parameters)**	**AIC**	R^2 **(PEF vs predictions)**
None (10)	1005719	0.931
Categorical (35)	1005718	0.931
Mixed (15)	1005680	0.932

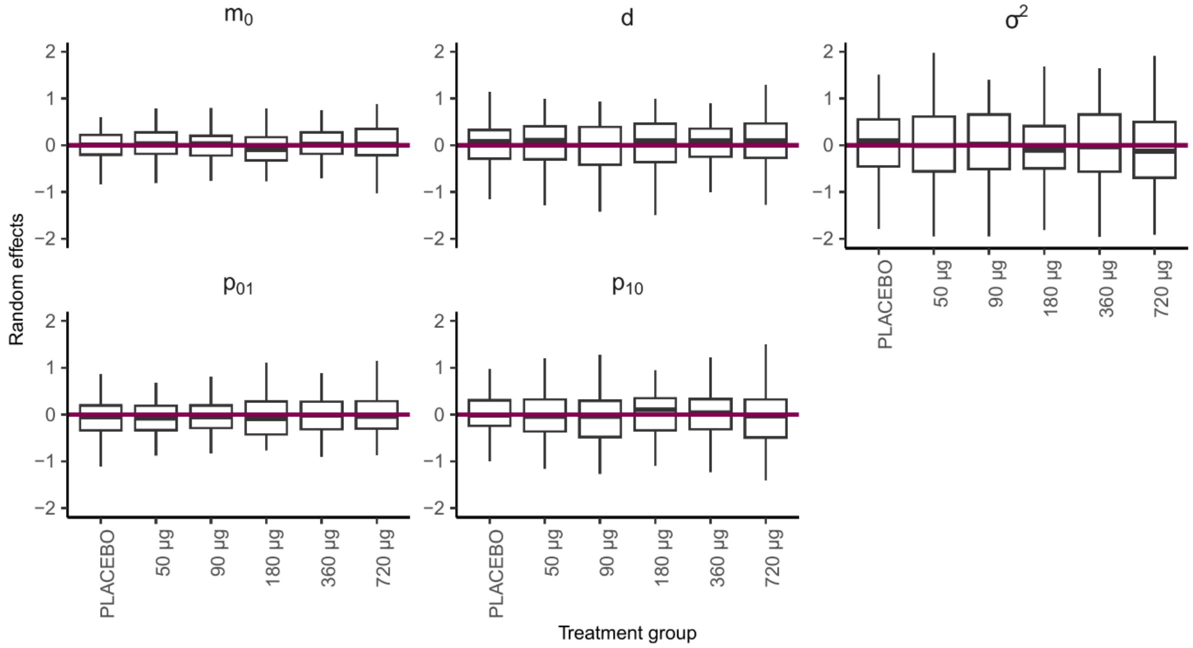

Figure S3: *Boxplots showing the distribution of estimated random effects from the model denoted "mixed", per model parameter and stratified on treatment group. The horizontal line is drawn at* $y = 0$.
